# Supplementary material for: Facile syntheses of conjugated polymers for photothermal tumour therapy
Source: Nat Commun. 2019 Mar 13;10:1192. doi: 10.1038/s41467-019-09226-6 (PMC6416255; doi:10.1038/s41467-019-09226-6)
Supplement: Supplementary file 1 — Supplementary Information [file 41467_2019_9226_MOESM1_ESM.pdf]

Supplementary Information

Facile syntheses of conjugated polymers for photothermal tumour therapy

Chen *et al*.

1. Supplementary figures

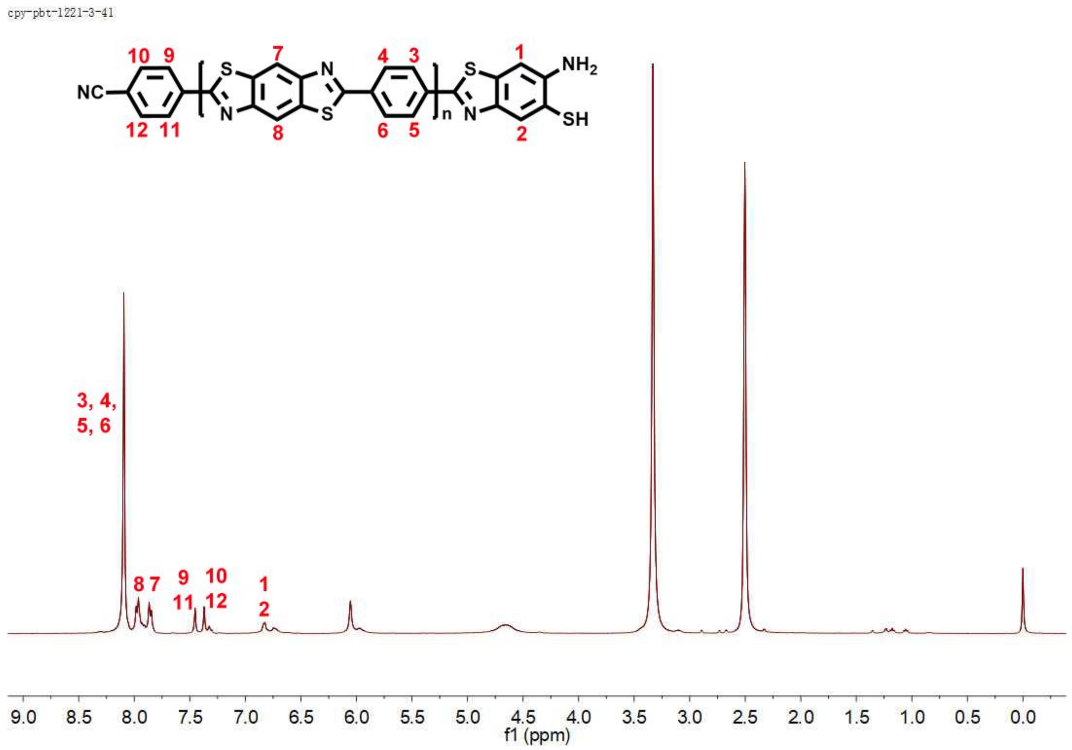

Supplementary Figure 1. ^{1}H NMR spectrum of PPBBT in DMSO-d_6.

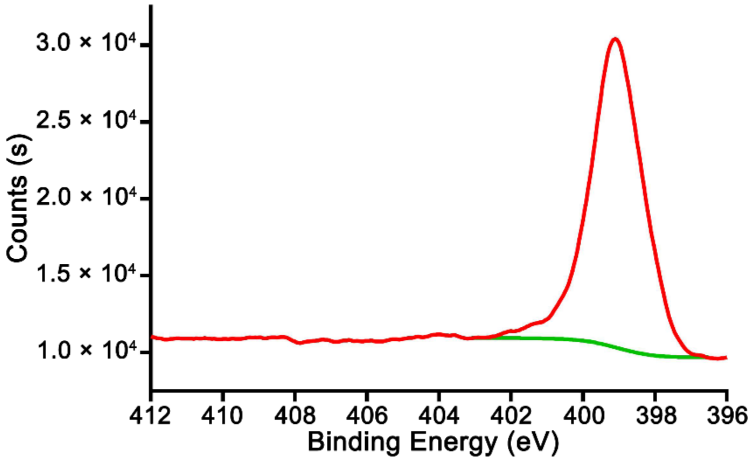

Supplementary Figure 2. XPS N 1*s* spectrum of PPBBT. The newly appeared peak at 398.7 eV verifies the formation of the newly formed C-N-C structures on the benzobisthiazole motif.

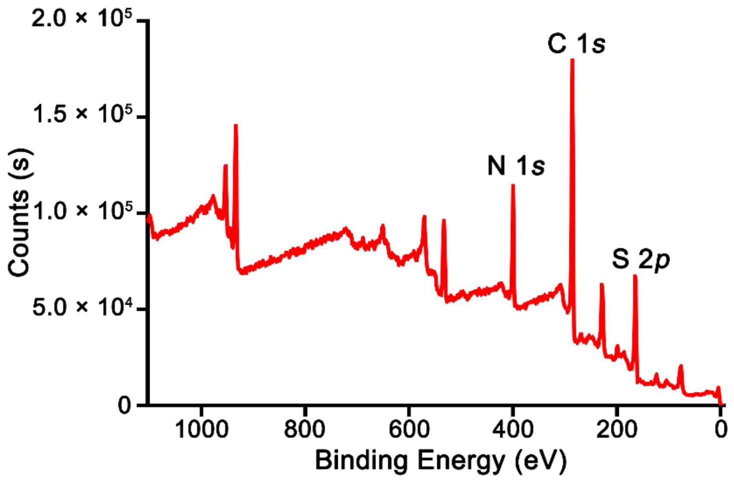

Supplementary Figure 3. XPS full spectrum of PPBBT for element composition analysis. Quantitative data are shown in Supplementary Table 1.

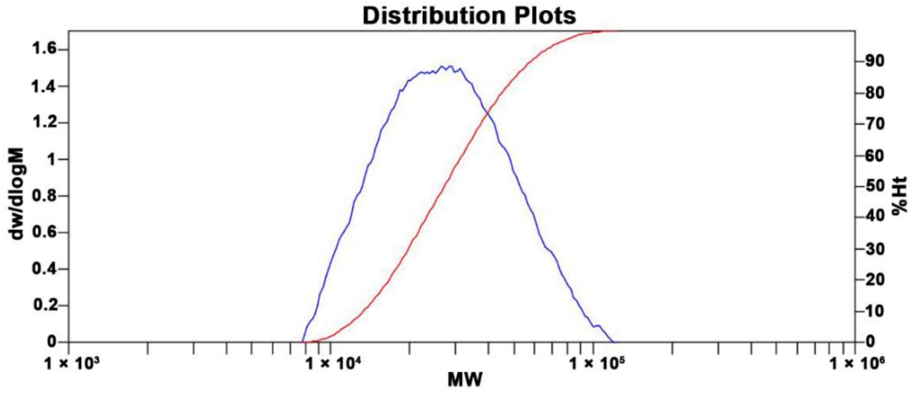

Supplementary Figure 4. GPC trace of PPBBT.

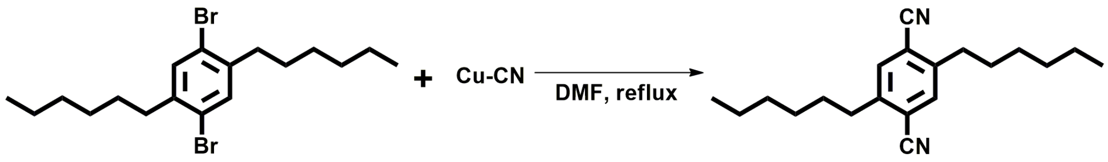

Supplementary Figure 5. Synthetic route for 2,5-dihexyl-1,4-dicyanobenzene.

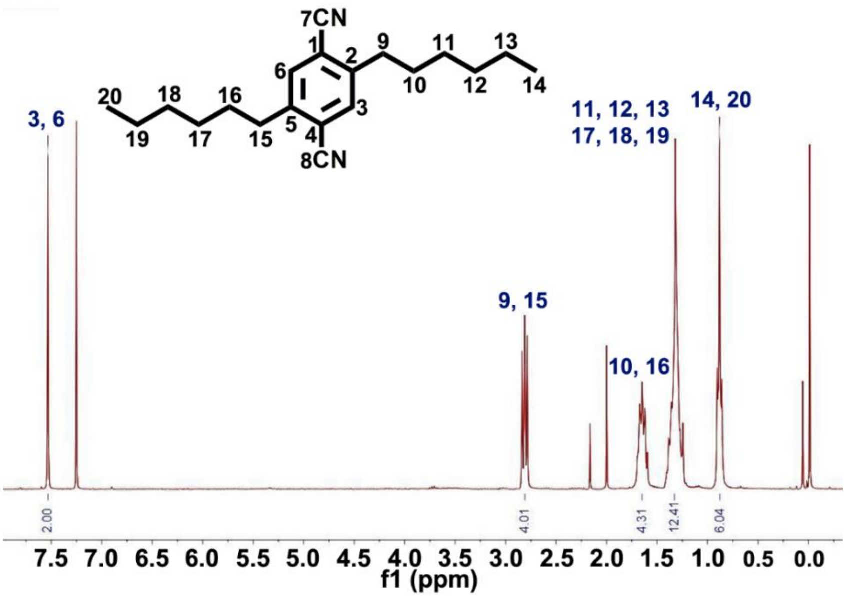

Supplementary Figure 6. ^{1}H NMR spectrum of 2,5-dihexyl-1,4-dicyanobenzene in $CDCl_3$.

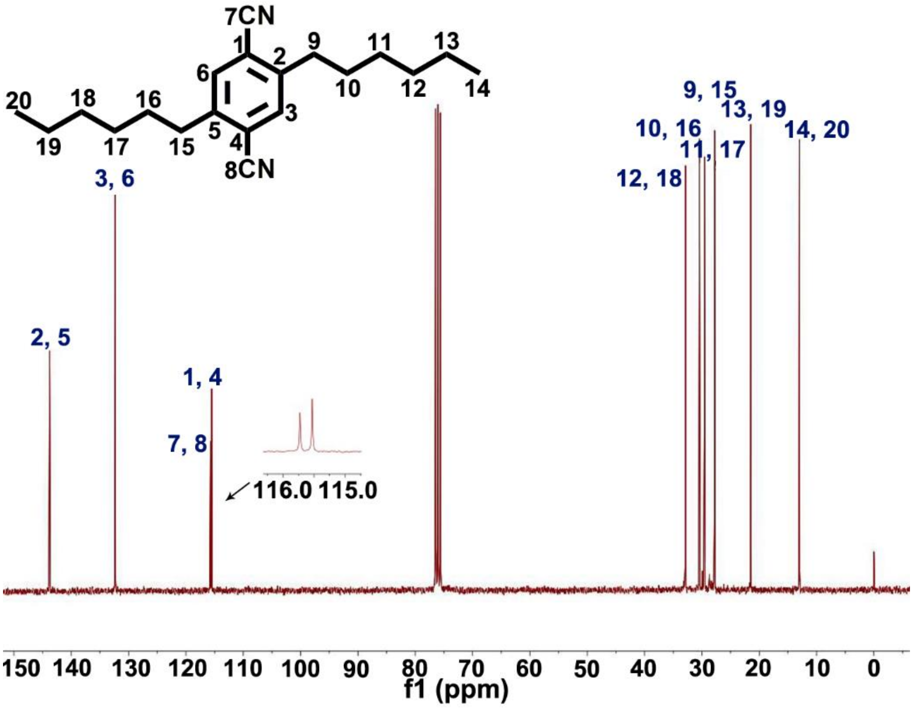

Supplementary Figure 7. ^{13}C NMR spectrum of 2,5-dihexyl-1,4-dicyanobenzene in $CDCl_3$.

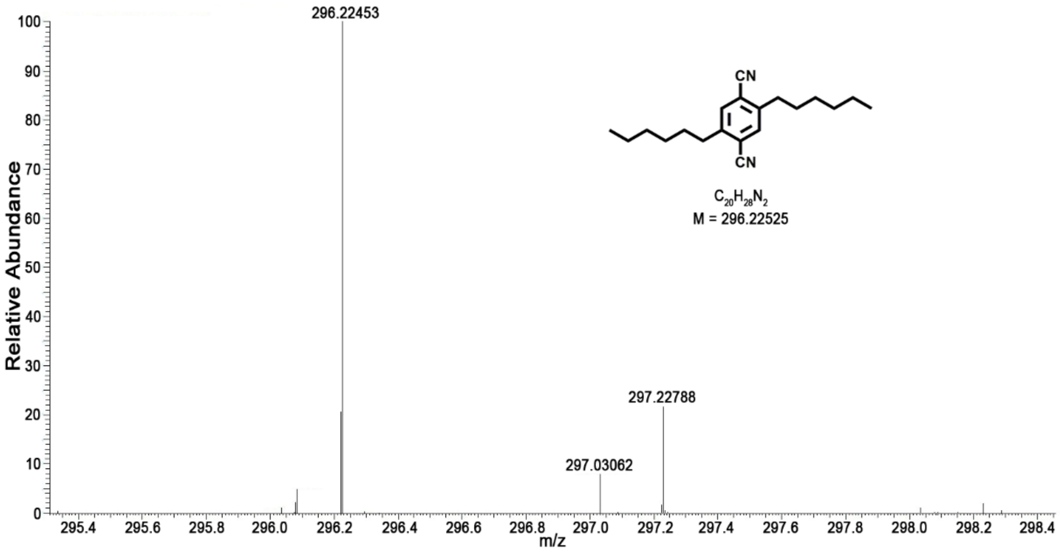

Supplementary Figure 8. EI-MS spectrum of 2,5-dihexyl-1,4-dicyanobenzene.

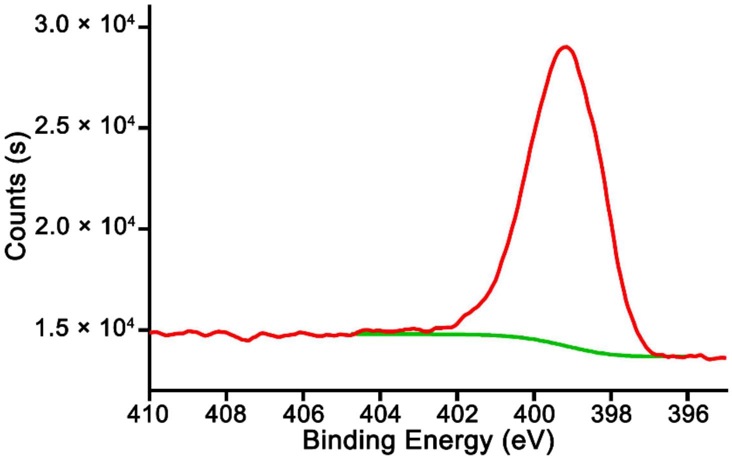

Supplementary Figure 9. XPS N 1*s* spectrum of Dihexyl-PPBBT. The newly appeared peak at 399.2 eV verifies the formation of the newly formed C-N-C structures on the benzobisthiazole motif.

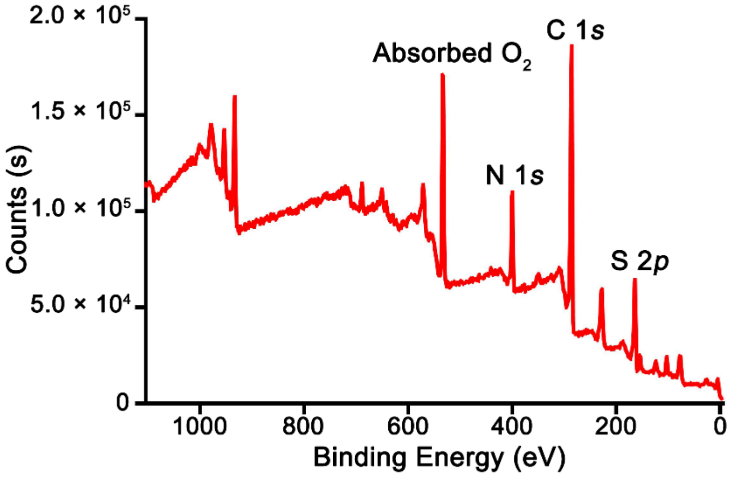

Supplementary Figure 10. XPS full spectrum of Dihexyl-PPBBT for element composition analysis. Quantitative data are shown in Supplementary Table 2.

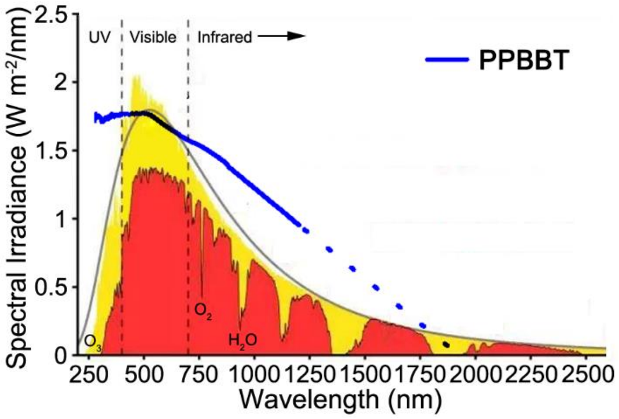

Supplementary Figure 11. UV-Vis-NIR spectrum of PPBBT merged with the irradiation spectrum of sunlight[1]. This graph shows the radiation spectrum for the direct light both at the top of the earth's atmosphere (yellow) and at sea level (red). The sun produces light with a distribution similar to that expected from a 5,250 °C blackbody (gray), which is approximately the sun's surface temperature. Cited and revised from Figure 2 of reference 1.

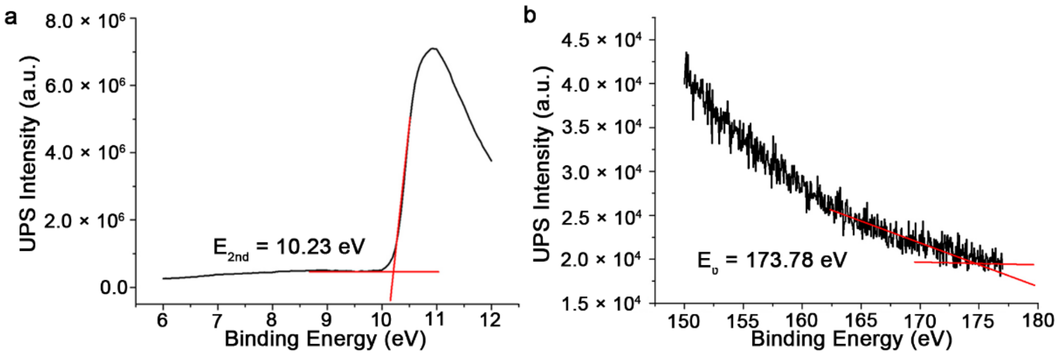

Supplementary Figure 12. Valence-band spectrum (**a**) and secondary electron cutoff (**b**) of PPBBT measured by UPS.

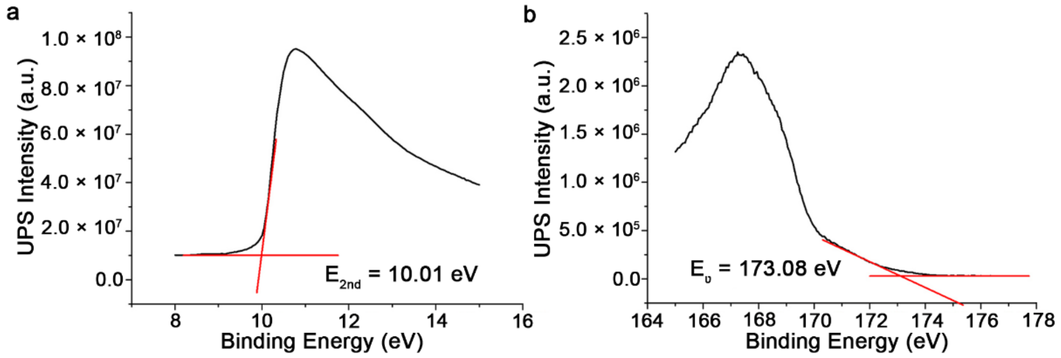

Supplementary Figure 13. Valence-band spectra (**a**) and secondary electron cutoff (**b**) of Dihexyl-PPBBT measured by UPS.

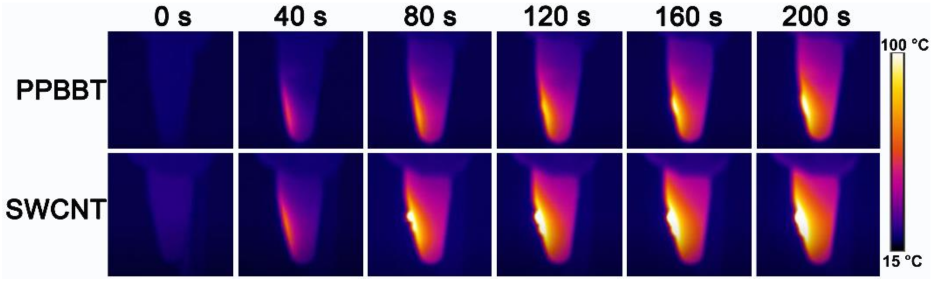

Supplementary Figure 14. Time-dependent thermal images of PPBBT and SWCNT powders in tubes exposed to 808 nm NIR laser at 1.5 W cm^{-2}.

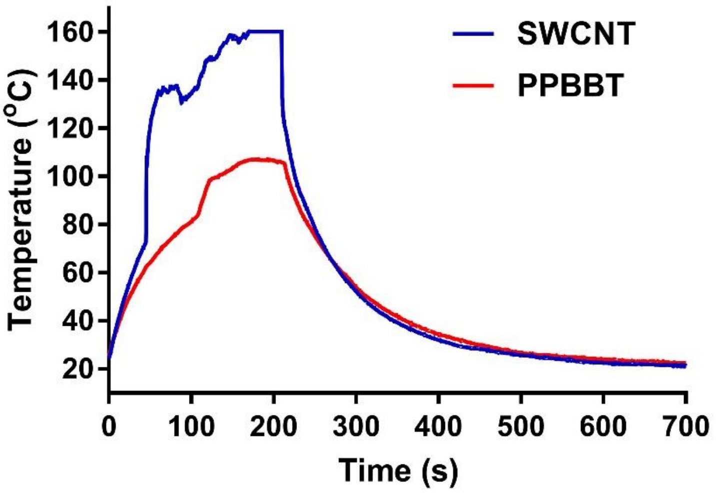

Supplementary Figure 15. Temperature curve of PPBBT and SWCNT powders as a function of laser irradiation time. The light source was removed at about 220 s.

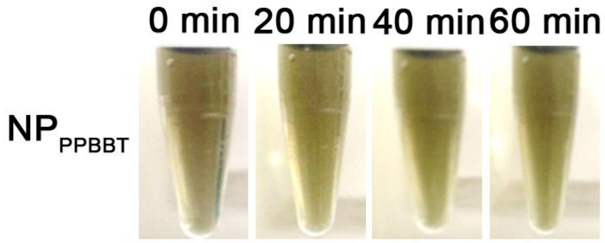

Supplementary Figure 16. Optical images of NP_{PPBBT} store for 0, 20, 40, and 60 min.

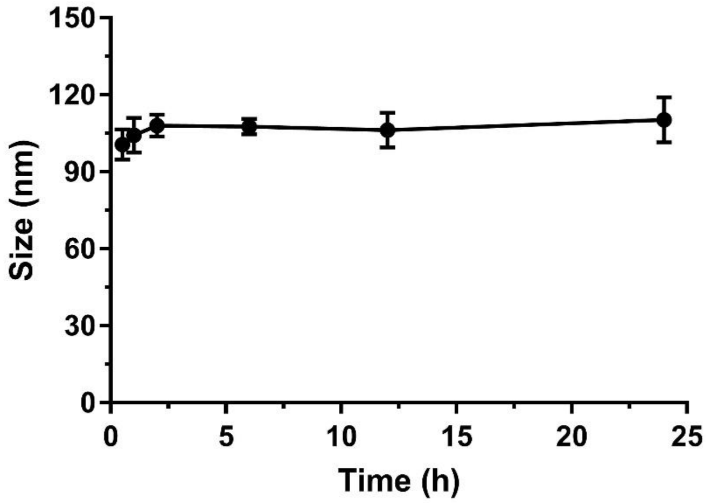

Supplementary Figure 17. Average diameter of NP_{PPBBT} store for different time measured by DLS. Results are presented as mean ± S.D., n = 3.

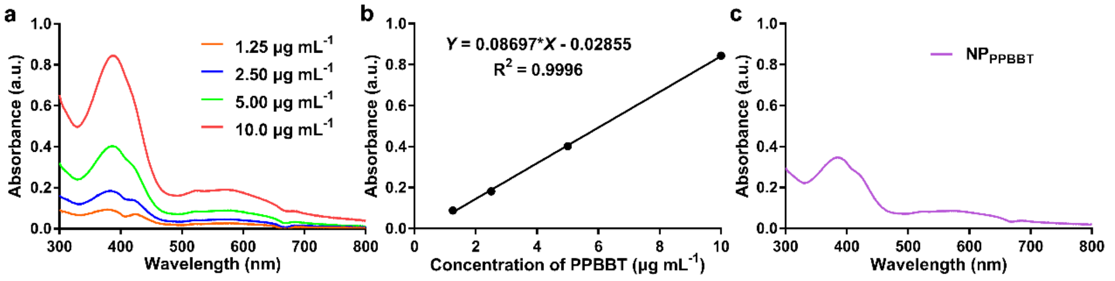

Supplementary Figure 18. Determination of the loading efficiency of PPBBT in NP_{PPBBT}. **a** UV-Vis spectra of PPBBT dissolved in DMSO at different concentrations. **b** Fitted calibration curve of UV-Vis absorbance *vs*. concentration of PPBBT in DMSO at 388 nm. **c** UV-Vis spectrum of as-prepared NP_{PPBBT} dissolved in DMSO (diluted 100 times). The concentration of PPBBT in the NP_{PPBBT} solution was calculated to be 4.30 μg mL^{-1}, thus the loading efficiency of PPBBT in NP_{PPBBT} was about 43% {(4.30 μg mL^{-1} * 100) / 1 mg mL^{-1} * 100% = 43%}.

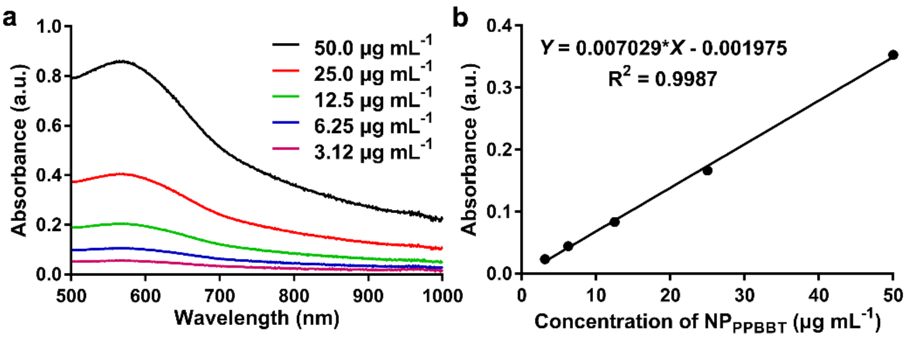

Supplementary Figure 19. Determination of the extinction coefficient of NP_{PPBBT}. **a** Vis-NIR spectra of NP_{PPBBT} in PBS at different concentrations. **b** Fitted calibration curve of Vis-NIR absorbance *vs*. concentration of NP_{PPBBT} in PBS at 808 nm.

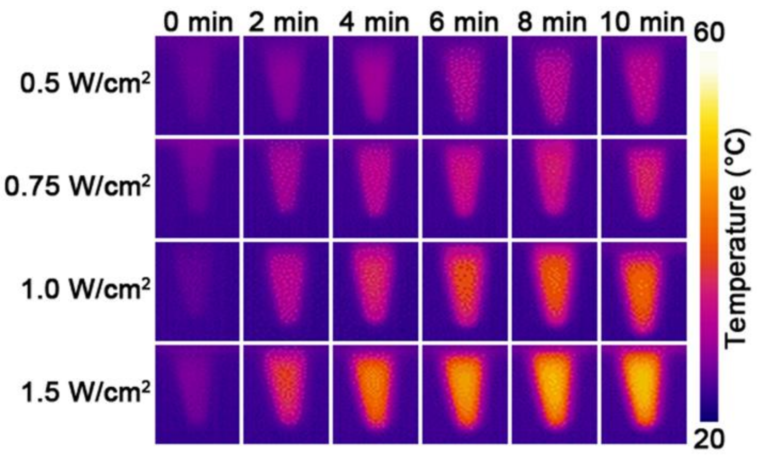

Supplementary Figure 20. Infrared thermal images of NP_{PPBBT} upon exposure to the NIR laser (808 nm, 10 min) at a power density of 0.5, 0.75, 1.0, or 1.5 W cm^{-2}.

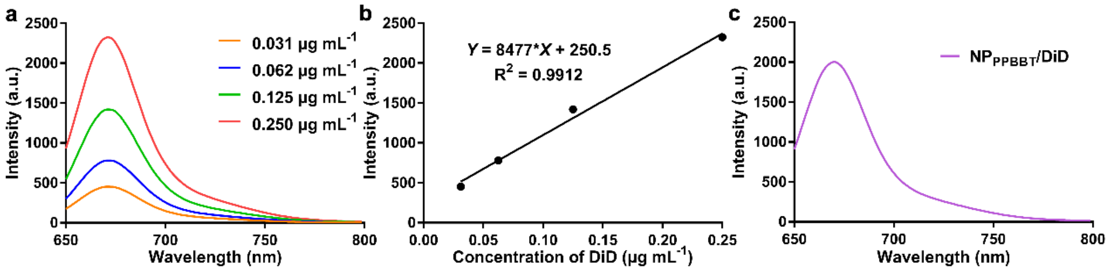

Supplementary Figure 21. Determination of the loading efficiency of DiD in NP_{PPBBT}/DiD. **a** Fluorescent spectra of DiD dissolved in DMSO at different concentrations. **b** Fitted calibration curve of fluorescence intensity *vs*. concentration of DiD in DMSO at 670 nm. **c** Fluorescent spectrum of NP_{PPBBT}/DiD dissolved in DMSO (diluted 100 times). The concentration of DiD in the NP_{PPBBT}/DiD solution was calculated to be 0.207 μg mL^{-1}, thus the loading efficiency of DiD in NP_{PPBBT}/DiD was about 83% {(0.207 μg mL^{-1} * 100) / 0.025 mg mL^{-1} * 100% = 83%}. Excitation: 633 nm.

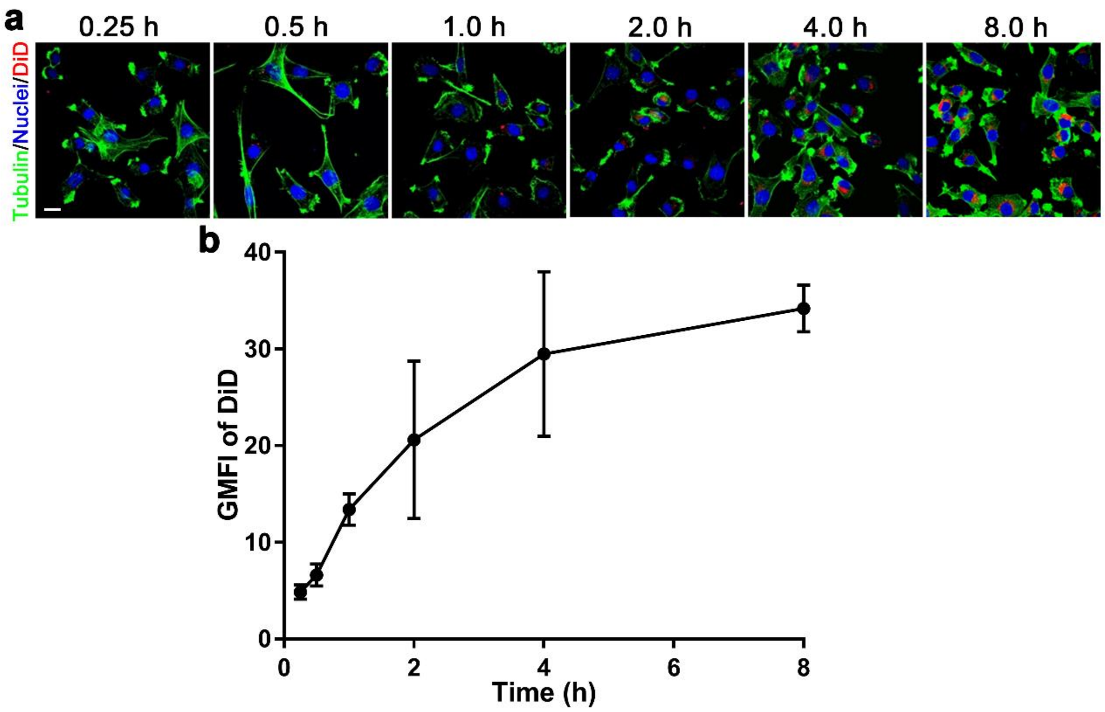

Supplementary Figure 22. Cell uptake of NP_{PPBBT}/DiD nanoparticle. **a** Confocal laser scanning microscopy fluorescence images of EMT-6 breast cells after incubation with NP_{PPBBT}/DiD nanoparticles for 0.25, 0.5, 1, 2, 4, or 8 h and then washed with PBS. Cell nucleus and cytoskeleton were stained by DAPI (blue) and Alexa Fluor 488 phalloidin (green), respectively. Scale bar, 20 μm. **b** Time course geometric mean fluorescence intensity (GMFI) of DiD in a. Results are presented as mean ± S.D., n = 3.

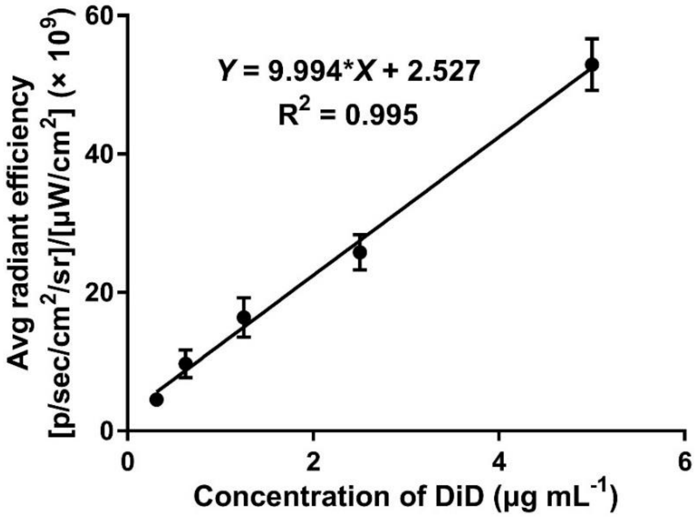

Supplementary Figure 23. Fitted calibration curve of the average radiant efficiency of DiD dissolved in fetal bovine serum at different concentrations. This curve was used to calculate the serum DiD concentration in Fig. 4a. Results are presented as mean ± S.D., n = 3.

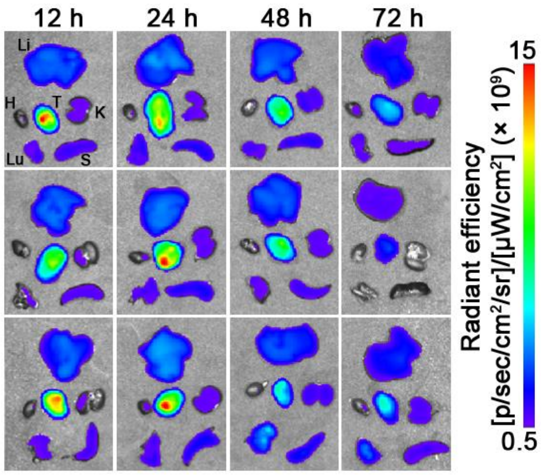

Supplementary Figure 24. *Ex vivo* DiD-fluorescent images of the major organs (heart (H), liver (Li), spleen (S), lung (Lu), kidney (K), and tumour (T)) excised from orthotopic EMT-6 tumour-bearing BALB/c mice at 12, 24, 48, or 72 h post *i.v.* injection of NP_{PPBBT}/DiD at a DiD dose of 0.25 mg kg^{-1} for each mouse (n = 3 for each time point).

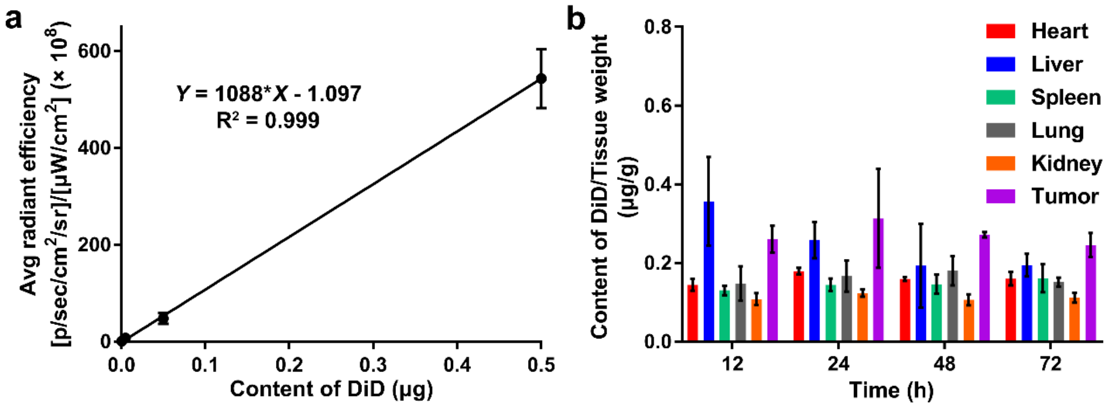

Supplementary Figure 25. Quantification of the content of DiD from the homogenized organs. **a** Fitted calibration curve of average radiant efficiency *vs*. content of DiD. **b** Quantification of the content of DiD (normalized by the tissue weight) from the homogenized organs (heart, liver, spleen, lung, kidney, and tumors) from Supplementary Figure 24. All organs were weighted and then homogenized in 1 mL 5% Triton X-100, respectively. Chloroform were used to extract DiD from homogenates for three times. The extracted DiD was redissolved in 500 μL methanol after the chloroform was evaporated, and the obtained solutions were sent for fluorescent quantification using Xenogen IVIS® spectrum system. The samples of the standard curve were treated in the same way of the organs. Results are presented as mean ±S.D., n = 3.

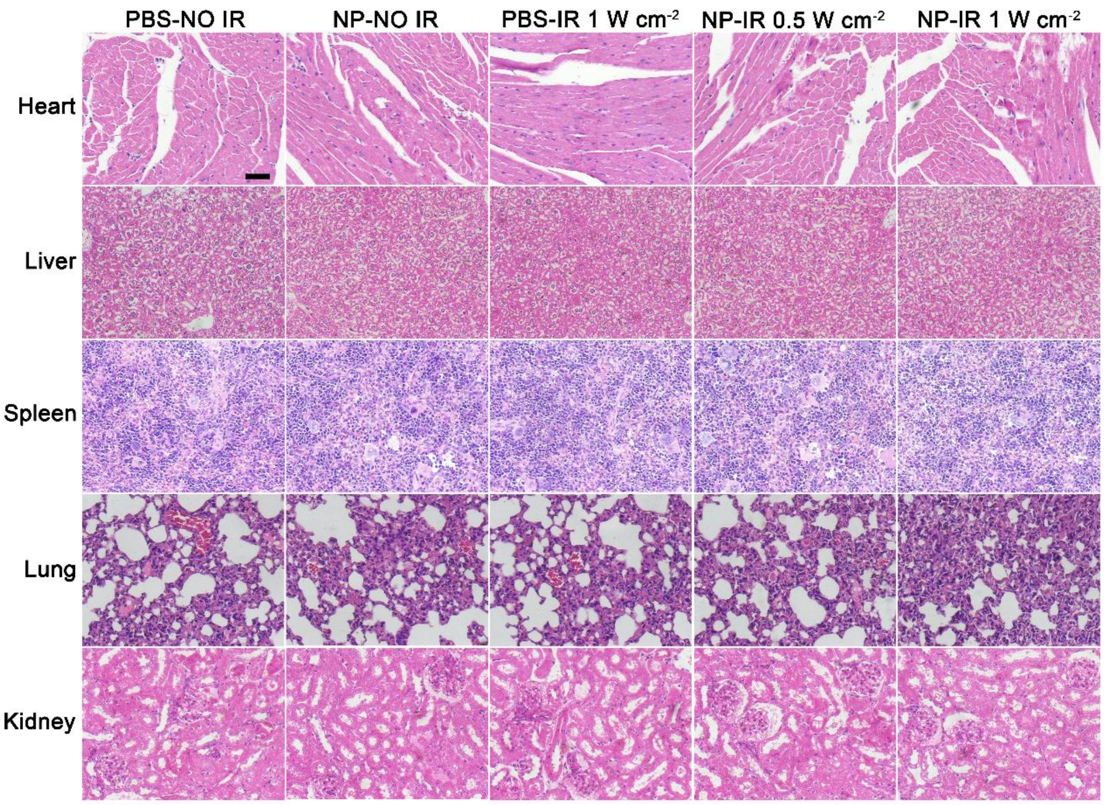

Supplementary Figure 26. H&E staining of the major organs (heart, liver, spleen, lung, and kidney) of mice sacrificed at day 15 post treatment. Scale bar, 50 μm.

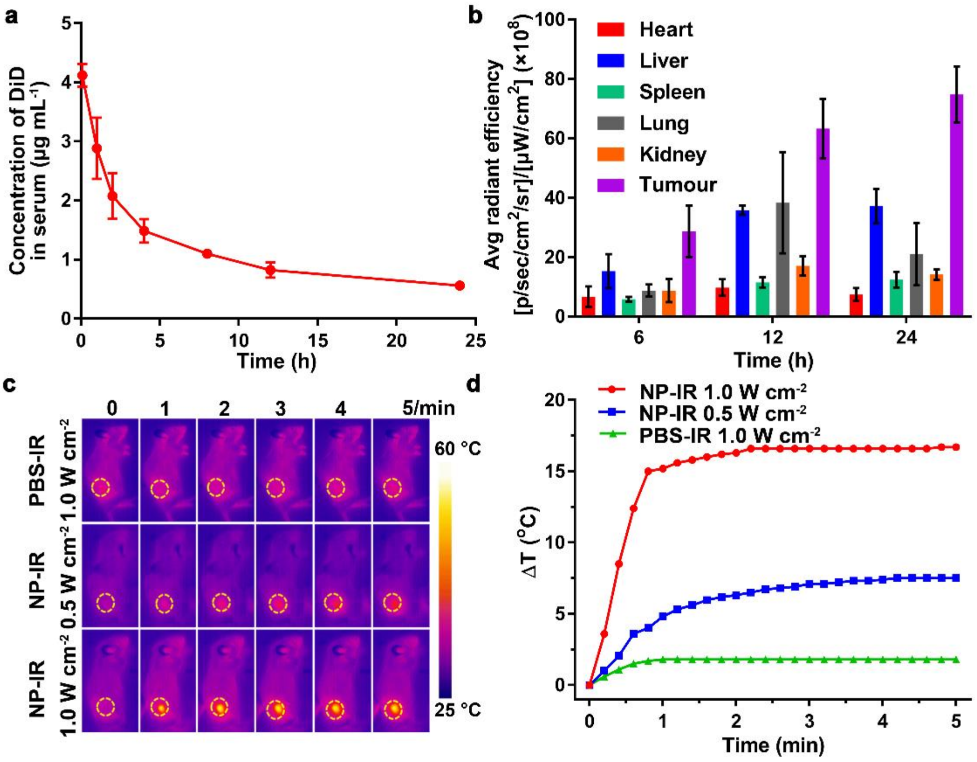

Supplementary Figure 27. *In vivo* performance of NP_{PPBBT} nanoparticles on tumours. **a** Blood DiD concentration *vs*. time curve in subcutaneous EMT-6 breast tumour-bearing mice intravenously injected with NP_{PPBBT}/DiD at a DiD dose of 0.25 mg kg^{-1}. **b** Quantification of DiD fluorescence from the major organs (heart, liver, spleen, lung, kidney, and tumours) in tumour-bearing mice sacrificed at 6, 12, or 24 h post injection of NP_{PPBBT}/DiD at a DiD concentration of 0.25 mg kg^{-1}. **c**, **d** IR thermal images (the tumours were indicated by yellow dashed circles) (**c**) and tumour temperature evolutions (**d**) of subcutaneous EMT-6 tumour-bearing mice at 12 h post *i.v.* injection of PBS or 5 mg kg^{-1} NP_{PPBBT} under 808 nm laser irradiation at 0.5 or 1.0 W cm^{-2} for 5 min. NP-IR: NP_{PPBBT} plus laser irradiation; PBS-IR: PBS plus laser irradiation. Results are presented as mean ± S.D., n = 3.

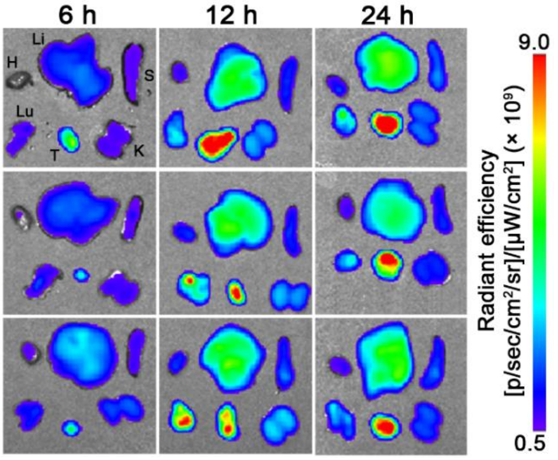

Supplementary Figure 28. *Ex vivo* DiD-fluorescent images of the major organs (heart (H), liver (Li), spleen (S), lung (Lu), kidney (K), and tumour (T)) excised from subcutaneous EMT-6 tumour-bearing BALB/c mice at 6, 12, or 24 h post *i.v.* injection of NP_{PPBBT}/DiD at a DiD dose of 0.25 mg kg^{-1} for each mouse (n = 3 for each time point).

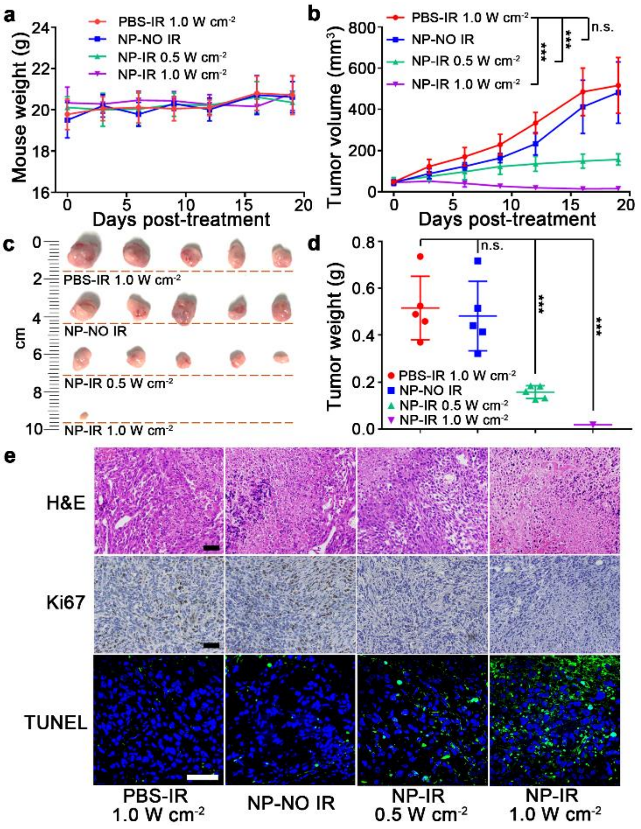

Supplementary Figure 29. PTT efficiency of NP_{PPBBT} nanopartiles on tumours. **a**, **b** Body weight (**a**) and tumour volume (**b**) curves of subcutaneous EMT-6 tumour-bearing mice at different time points after receiving one dose of treatment with indicated formulations. At 12 h post injection, tumours were irradiated with (or w/o) laser for 10 min and the observations started. **c**, **d** Photographs (**c**) and weights (**d**) of tumours in mice sacrificed after 18-day observation. **e** H&E staining, Ki67 staining (brown: Ki67-positive nuclei, blue: Ki67-negative nuclei), and TUNEL (FITC, green) and DAPI (blue) counterstaining of the tumour tissues from mice sacrificed at day 18 post treatment. Scale bar, 50 μm. PBS-IR: PBS plus laser irradiation; NP-IR: NP_{PPBBT} plus laser irradiation; NP-NO IR: NP_{PPBBT} without laser irradiation. Results are presented as mean ± S.D., n = 5; n.s., not significant, $p > 0.05$; *$p < 0.05$; **$p < 0.01$; ***$p < 0.001$, analyzed by Student's t test.

2. Supplementary Tables

Supplementary Table 1. Element composition analysis of the XPS spectrum of PPBBT in Supplementary Figure 3.

Element	At. %
C 1*s*	67.0
S 2*p*	16.3
N 1*s*	16.7

Supplementary Table 2. GPC trace analysis of PPBBT in Supplementary Figure 4.

MW Averages

Peak No	Mp	Mn	Mw	Mz	Mz + 1	Mv	PDI
1	28,986	23,176	31,089	41,496	52,796	29,717	1.34

Processed Peaks

Peak No	Start RT (mins)	Max RT (mins)	End RT (mins)	Pk Height (mV)	%Height	Area (mV.secs)	%Area
1	13.12	14.15	15.02	-1.99685	100	125.849	100

Supplementary Table 3. Element composition analysis of the XPS spectrum of Dihexyl-PPBBT in Supplementary Figure 10.

Element	At. %
C 1*s*	71.7
S 2*p*	15.0
N 1*s*	13.3

Supplementary Table 4. Pharmacokinetic parameters of NP_{PPBBT}/DiD nanoparticels intravenously adminstered to the healthy mice or orthotopic EMT-6 tumour-bearing mice in Fig. 4a. The data were obtained by noncomparement analysis (DAS 3.2.6).

Parameter	C_{max} (μg L^{-1})	$T_{1/2z}$ (h)	$AUC_{0\text{-}72\ h}$ (μg L^{-1} h)	CLz (L h^{-1} kg^{-1})
Healthy mice	92.8	95.4	3131.6	0.716
Orthotopic EMT-6 tumour-bearing mice	88.6	46.6	2018.6	1.73

C_{max}, Peak concentrarion; $T_{1/2z}$, Elimination half life;
AUC, Area under the curve; CLz, Clearance rate.

Supplementary Table 5. HPLC condition for the purification of 2,5-dihexyl-1,4-dicyanobenzene.

Time (min)	Flow (mL min^{-1})	H_2O% (0.1% TFA)	CH_3CN% (0.1% TFA)
0	12.0	20	80
3	12.0	20	80
35	12.0	0	100
37	12.0	0	100
38	12.0	20	80
40	12.0	20	80

3. Supplementary Reference

1 Tanaka, Y., Matsuo, K. & Yuzuriha, S. Long-lasting muscle thinning induced by infrared irradiation specialized with wavelengths and contact cooling: a preliminary report. *Eplasty* **10**, 327-335 (2010).
